# Factors associated with excessive polypharmacy in older people

**DOI:** 10.1186/s13690-015-0095-7

**Published:** 2015-11-09

**Authors:** Denise Walckiers, Johan Van der Heyden, Jean Tafforeau

**Affiliations:** Scientific Institute of Public Health, Operational Direction Public Health and Surveillance, Unit Surveys, Lifestyle and Chronic Conditions, Brussels, Belgium

**Keywords:** Polypharmacy, Multiple medication/medicine/drug use, Elderly, Aged, General population, Health interview survey, Belgium

## Abstract

**Background:**

Older people are a growing population. They live longer, but often have multiple chronic diseases. As a consequence, they are taking many different kind of medicines, while their vulnerability to pharmaceutical products is increased. The objective of this study is to describe the medicine utilization pattern in people aged 65 years and older in Belgium, and to estimate the prevalence and the determinants of excessive polypharmacy.

**Methods:**

Data were used from the Belgian Health Interview Survey carried out in 2008. Each respondent was asked to show to the interviewer all medicines that he/she had taken in the 24 h prior to the interview. Excessive polypharmacy was defined as the use of nine different kind of medicines or more in the past 24 h; the relation with the Region of residence, age, gender and additional factors, such as socioeconomic status, living situation, health status and contacts with health services, was explored through multivariate models.

**Results:**

Eight percent of the older people (65 years or more) belong to the excessive polypharmacy group. Factors most strongly associated with excessive polypharmacy are: having a longstanding illness, chronic condition or handicap, at least 1 contact with a general practitioner in past 2 months and self-reported depression during the last year. Ninety percent of persons in the excessive polypharmacy group are taking medicines active on the cardiovascular system.

**Conclusions:**

In order to optimize the use of medicines, it is necessary to find a balance between adequate treatment of diseases and avoiding adverse effects of medicines. Interventions should aim to increase awareness among healthcare professionals and patients; they should focus on general practitioners and patients with cardiovascular diseases, those suffering from depression and those aged 80 years and over. Monitoring excessive polypharmacy in the older population remains important. Further studies should explore more in depth other and more specific determinants of excessive polypharmacy.

## Background

Older people are a growing population. They live longer, but often have multiple chronic diseases and take many different kind of medicines, while their vulnerability to pharmaceutical products is increased. There is also an increase in the availability of medicines on the market and, due to the development of generic products, an easier access to medicines.

There is no consensus in the literature on the definition of the concept of polypharmacy and excessive polypharmacy. Polypharmacy can for instance either be defined by a single count of medicines - frequently five or more medicines used concomitantly [1], or by the consumption of more medicines than clinically indicated or by the consumption of medicines that are not clinically indicated [2]. The WHO defines polypharmacy as “the administration of many drugs at the same time or the administration of an excessive number of drugs” [3]. Excessive polypharmacy (*EPP*) is defined as the concomitant use of nine/ten or more medicines taken regularly or as-needed [4–12].

The way in which the medicine use is measured varies also from one study to the other: it can involve prescribed and/or over-the-counter (*OTC*) medicines, vaccines, magistral preparations, herbal preparations, vitamins, minerals, dietary supplements, alternative medicines (homeopathic medicines). They can be taken on a regular basis and “as-needed”, one-time-only, for systemic or local use.

There are several risks associated with medicine use, such as adverse effects, medicine-medicine and medicine-disease interactions, decreasing adherence to medicine therapy or errors in the actual use of the medicines. This leads also to unnecessary health costs, related to medicines “prescribing cascade”, medical consultations and hospitalizations.

It is consequently important to monitor excessive polypharmacy in the population and explore determinants that contribute to it. In Europe, some studies already explored this, but most of them consider only home care patients [4] or nursing home patients [8, 10] or are limited to a specific region [12, 13]. The present study explores within a representative sample of the general population in Belgium, the actual use of medicines in people aged sixty-five years and older, taking into account socio-demographic information, health related information and health care consumption.

## Methods

### Study population

Data were used from the Belgian Health Interview Survey carried out in 2008. Information was collected through a face-to-face interview and a self-completion questionnaire from a sample of 11,254 individuals, which is representative of the population living in Belgium. A stratified, clustered multistage sampling design was implemented for this survey [14]. The analyses for the current study were restricted to the population of 65 years and older (*N* = 2835) with both community-based and institutionalized persons included.

### Definition

Extensive information was gathered on the use of medicines. Each respondent was asked to show to the interviewer all medicines that he/she had taken in the 24 h prior to the interview. The brand name and the national identification code (unique code for each registered medicine in Belgium) was recorded by the interviewer and subsequently coded with the Anatomical Therapeutic Chemical (*ATC*) classification system of the World Health Organization (version 10 December 2009^1^). Drug groups were taken into account until the 5th level of the ATC classification. A combination medicine (multiple component products) was considered as a single medicine. The dosage was not taken into account. Medicines with different brand names and generics having the same ATC-code were considered as one medicine.

The study considered medicines taken in the 24 h prior to the interview, including both those taken on a regular basis and those taken “as needed”. It was limited to official medicines that were used in an ambulatory setting, for systemic or local use, and listed in the Annotated Directory of Medicines published by the Belgian Centre for Pharmacotherapeutic Information (*BCPI*; [15]). They could be prescribed or OTC medicines. Excessive polypharmacy was defined here as the use of nine different medicinal products or more in the past 24 h.

### Statistical analysis

The use of medicines was explored in relation to potential determinants according to the literature. Apart from Region of residence, age and gender, several socioeconomic variables were considered, such as educational attainment (defined at the level of the household), equivalent household income, living or not in an institution for elderly, health status (longstanding illnesses, chronic conditions or handicaps, depression that has lasted for at least 2 weeks during the past 12 months), contacts in the past 2 months either with a general practitioner (*GP*) or with a specialist, and hospitalization in the past 12 months.

In a first step a univariate analysis was conducted to describe the polypharmacy level in the study sample; three groups were considered: non polypharmacy (0–4 medicines), polypharmacy (5–8 medicines) and EPP (at least 9 medicines). In a second step, the relation between polypharmacy and its determinants, but also between polypharmacy and the use of specific group of medicines was explored in the bivariate analyses. Differences in the bivariate analyses were assessed with Chi-square tests.

Multivariate analyses were used to assess the association between specific groups of medicines and potential determinants. Determinants of polypharmacy were explored in a multinomial logistic regression model.

Statistical analyses were performed with SAS®9.3, taking into account the sampling design of the survey.

## Results

The mean number of medicines used per person aged 65 years and over is 3.5 (range: 0–19). Almost one fifth of the population (18.1 %; 95 % CI: 15.8–20.4) did not take any medicine during the past 24 h, 48.9 % (95 % CI: 46.1–51.7) took 1 to 4 medicines; 24.8 % (95 % CI: 22.4–27.1) belongs to the polypharmacy group (Table 1) and 8.2 % (95 % CI: 6.3–10.1) to the excessive polypharmacy group. When we consider only persons living in an institution for elderly (*N* = 313), the mean number of medicines used per person is 4.5 (range: 0–18) and the percentage in the excessive polypharmacy group reaches 16.8 % (95 % CI: 11.2–22.3). For persons living in the community (*N* = 2507), these figures are respectively 3.5 (range 0–19) and 7.7 % (95 % CI: 5.7–9.8).

**Table 1:** Characteristics of the population of 65 years and older, in percentage, Belgium, 2008

Characteristic	Total mean: 3.5		0–4 medicines (*N* = 1769) mean: 1.7		5-8 medicines (*N* = 811) mean: 6.1		> = 9 medicines [9–19] (*N* = 255) mean: 10.6	
	%	95 % CI	%	95 % CI	%	95 % CI	%	95 % CI
*N* = 2835	100.0		67.0	64.4–69.7	24.8	22.4–27.1	8.2	6.3–10.1
Demographics (*N* = 2835)								
Gender								
Males	41.2	39.1–43.3	43.5	40.7–46.3	37.6	32.9–42.3	33.4	19.3–47.5
Females	58.8	56.7–60.9	56.5	53.7–59.3	62.4	57.7–67.0	66.6	52.5–80.7
Age								
65–69	23.2	20.5–25.9	26.8	23.3–30.2	17.2	13.0–21.4	12.2	5.5–18.9
70–74	27.2	24.1–30.3	27.9	24.3–31.5	24.8	19.5–30.0	28.7	13.4–44.0
75–79	19.4	17.1–21.7	19.4	16.5–22.3	20.0	16.1–24.0	17.1	10.1–24.1
> = 80	30.2	27.7–32.8	25.9	23.0–28.9	38.0	33.1–42.8	42.0	31.1–52.9
Socioeconomic status								
Education (*N* = 2689)								
No degree/Primary	30.4	27.3–33.4	27.6	24.1–31.1	35.0	29.9–40.0	38.9	27.4–50.4
Lower secondary	24.4	21.0–27.7	22.4	18.8–26.0	26.3	21.1–31.4	34.9	20.1–49.8
Higher secondary	27.0	23.8–30.2	29.2	25.3–33.1	23.9	19.1–28.8	18.4	10.3–26.5
Higher education	18.2	15.6–20.9	20.8	17.5–24.0	14.8	10.6–19.0	7.8	3.2–12.3
Equivalent household income (*N* = 2271)								
Quintile 1	20.5	17.2–23.8	19.6	16.2–23.0	17.6	13.6–21.6	37.1	20.7–53.6
Quintile 2	32.8	29.2–36.4	32.5	28.2–36.9	34.0	28.4–29.6	31.0	19.8–42.3
Quintile 3	25.4	21.9–28.8	24.2	20.1–28.3	30.2	24.5–35.8	20.3	11.1–29.5
Quintile 4	11.2	9.1–13.4	11.7	9.0–14.4	10.6	7.2–14.0	9.0	3.7–14.4
Quintile 5	10.1	7.9–12.2	11.9	9.2–14.7	7.6	4.2–11.0	2.5	0.2–4.8
Living situation (*N* = 2820)								
Living alone or with other person(s) in home setting	94.4	93.4–95.4	95.9	94.8–96.9	92.3	90.3–94.4	88.6	84.0–93.2
Living in a residential home for the elderly	5.6	4.6–6.6	4.1	3.1–5.2	7.6	5.6–9.7	11.4	6.8–16.0
Region (*N* = 2835)								
Flemish Region	61.2	58.9–63.4	66.2	63.4–68.9	50.8	45.8–55.8	51.8	39.8–63.9
Brussels Region	8.8	8.1–9.5	8.5	7.6–9.5	9.7	8.0–11.4	7.8	4.6–10.9
Walloon Region	30.1	28.0–32.1	25.3	22.7–27.9	39.5	34.7–44.3	40.4	29.6–51.1
Health status								
Longstanding illness, chronic conditions or handicaps (*N* = 2819)								
Yes	54.0	51.0–56.9	42.3	38.7–45.9	73.4	69.0–77.9	90.2	85.0–95.4
No	46.0	43.1–49.0	57.7	54.1–61.3	26.6	22.1–31.0	9.8	4.6–15.0
Depression during the past 12 months (*N* = 2794)								
Yes	6.8	4.9–8.6	4.2	2.8–5.5	8.0	5.6–10.4	24.4	8.9–39.9
No	93.2	91.4–95.1	95.8	94.4–97.2	92.0	89.6–94.4	75.6	60.1–91.1
Contact with health services								
Contact with GP in past 2 months (*N* = 2811)								
Yes	73.8	71.1–76.4	65.4	61.8–69.0	89.4	86.4–92.5	94.1	89.9–98.3
No	26.2	23.6–28.9	34.6	31.0–38.2	10.6	7.4–13.6	5.9	1.7–10.1
Contact with specialist in past 2 months (*N* = 2817)								
Yes	27.8	25.3–30.3	24.8	21.6–27.9	33.3	28.2–38.4	36.3	25.6–47.0
No	72.2	69.7–74.7	75.2	72.1–78.3	66.7	61.6–71.8	63.7	53.0–74.4
Inpatient hospitalization in past 12 months (*N* = 2813)								
Yes	18.2	16.0–20.4	14.0	11.5–16.5	23.5	19.4–27.6	36.1	25.1–47.1
No	81.8	79.6–84.0	86.0	83.5–88.5	76.5	72.4–90.6	63.9	52.9–74.9
1 day patient hospitalization in past 12 months (*N* = 2807)								
Yes	11.0	9.1–12.9	10.2	7.9–12.6	13.4	9.5–17.4	10.3	5.0–15.5
No	89.0	87.1–90.9	89.8	87.4–92.1	86.6	82.6–90.5	89.7	84.5–95.0

### Description of the study population by level of polypharmacy

#### Socio-demographic characteristics

The mean age of the study population is 75.5 years (range: 65–105). There are 58.8 % of women in the study group (Table 1).

The age distributions are significantly different between the “non-polypharmacy” group and the polypharmacy groups, but not between the two polypharmacy groups.

In the EPP-group, only 7.8 % has a higher education and 2.5 % has a higher household income, versus 14.8 and 7.6 % respectively in the polypharmacy group, and 20.8 and 11.9 % respectively in the “non-polypharmacy” group.

In the two polypharmacy groups, the percentage of persons living in a residential home for the elderly (respectively 7.6 % in the polypharmacy group and 11.4 % in the EPP-group) is higher than in the “non-polypharmacy” group (4.1 %), but there is no significant difference between the two polypharmacy groups. The same is observed when only the persons aged 80 and older are considered with respectively 18.3 % (95 % CI: 13.6–23.0) and 21.1 % (95 % CI: 13.5–28.7) institutionalized people in the polypharmacy and EPP-groups versus 11.8 % (95 % CI: 8.8–14.9) in the non-polypharmacy group.

In the two polypharmacy groups, the percentage of persons living in the Flanders Region is lower than in the “non-polypharmacy” group (and the opposite is observed for the persons living in the Wallonia Region), but there is no difference between the two polypharmacy groups.

#### Health status and use of health care characteristics

Suffering from a longstanding illness, chronic condition or handicap is associated with (excessive) polypharmacy (Table 1); in addition there is a difference between the polypharmacy (73.4 % of users) and the EPP (90.2 % of users) groups. 24.4 % of persons in the EPP-group were suffering from depression in past 12 months versus 8.0 and 4.2 % respectively in the polypharmacy and “non-polypharmacy” groups.

Recent contacts with health professionals are associated with (excessive) polypharmacy: 94.1 % of persons in the EPP-group had a contact with a GP in the last two months and 36.3 % with a specialist (versus respectively 65.4 and 24.8 % in the “non-polypharmacy” group), but there is no difference between the two polypharmacy groups.

At least one inpatient hospitalization in past 12 months is associated with (excessive) polypharmacy (with in addition a difference between the polypharmacy and the EPP groups), while at least one day patient hospitalization in past 12 months is not associated with (excessive) polypharmacy.

### Use of specific groups of medicines by level of polypharmacy

Table 2 presents the percentage of the population that used medicines from a particular anatomical group (first level of the ATC classification) for groups of medicines used by at least 10 % of the persons: medicines active on the cardiovascular system are the most frequently used drugs, followed by the drugs acting on the nervous system. The percentages of use of the groups vary according to polypharmacy status but the ranking is the same. Except for cardiovascular medicines, there is also a difference between the polypharmacy group and the EPP group. Among people in the polypharmacy groups, more than 90 % use cardiovascular medicines. The use of medicines acting on the nervous system occurs among 86.5 % of the people in the EPP group and 65.9 % of those in the polypharmacy group.

**Table 2:** Percentage of population (65 years and older) using medicines, in function of the anatomical groups^a^ (ATC_1), according to polypharmacy status (*N* = 2835), Belgium, 2008

ATC_1		Total		0–4 medicines (*N* = 1769)		5–8 medicines (*N* = 811)		> = 9 medicines (*N* = 255)	
		%	95 % CI	%	95 % CI	%	95 % CI	%	95 % CI
C	Cardiovascular system	61.9	59.1–64.7	46.6	43.1–50.2	92.2	89.5–94.9	95.3	91.7–98.8
N	Nervous system	37.6	34.7–40.6	21.2	18.3–24.0	65.9	61.0–70.7	86.5	79.6–93.3
A	Alimentary tract and metabolism	33.5	30.7–36.4	18.3	15.5–21.2	56.5	51.3–61.7	88.0	82.7–93.3
B	Blood and blood forming organs	31.2	28.5–34.0	17.2	14.5–19.9	54.9	49.8–59.9	74.3	65.7–82.9
M	Musculo-skeletal system	18.1	15.7–20.5	11.5	9.0–14.0	25.2	20.7–29.7	50.6	38.5–62.7
R	Respiratory system	12.1	9.9–14.3	5.1	3.5–6.6	20.8	16.1–25.5	42.9	29.6–56.2

^a^for those used by at least 10 % of the persons

Table 3 presents the percentage of the population that used medicines from a particular pharmacological class (ATC_3) by level of polypharmacy for the ten most frequently used pharmacological subgroups. Antithrombotic agents are the most commonly used pharmacological class. The use of these drugs is particularly high among people with EPP.

**Table 3:** Percentage of population (65 years and older) using medicines, in function of pharmacological classes^a^ (ATC_3), according to polypharmacy status (*N* = 2835), Belgium, 2008

ATC_3		Total		0-4 medicines (*N* = 1769)		5-8 medicines (*N* = 811)		> = 9 medicines (*N* = 255)	
		%	95 % CI	%	95 % CI	%	95 % CI	%	95 % CI
B01A	Antithrombotic agents	30.5	27,8–33,3	17.0	14.2–19.7	53.3	48.2–58.4	72.5	63.6–81.4
C10A	Lipid modifying agents, plain	27.9	25,0–30,7	18.7	15.8–21.7	44.8	39.7–50.0	51.3	39.3–63.3
C07A	Beta blocking agents	24.2	21,4–26,9	13.7	11.3–16.1	42.2	37.2–47.2	55.2	43.6–66.7
A02B	Drugs for peptic ulcer and gastro–oesophageal reflux disease	14.9	12,6–17,1	6.2	4.5–8.0	25.7	21.1–30.4	52.4	40.5–64.2
N05B	Anxiolytics	13.4	11,3–15,6	6.1	4.5–7.6	22.0	17.7–26.4	47.4	34.9–59.9
C08C	Selective calcium channel blockers with mainly vascular effects	12.7	10,4–15,0	6.8	4.8–8.8	21.5	16.9–26.1	34.0	19.7–48.4
C09A	Ace inhibitors, plain	11.4	9,6–13,2	6.2	4.2–8.2	21.8	17.6–25.9	22.0	14.5–29.6
N06A	Antidepressants	10.9	9,2–12,5	3.3	2.2–4.3	23.2	18.6–27.9	35.5	25.1–45.8
M01A	Antiinflammatory and antirheumatic products, non-steroids	10.3	8,2–12,4	6.7	4.9–8.5	13.5	9.7–17.2	29.8	14.9–44.8
N05C	Hypnotics and sedatives	10.2	8,4–12,0	5.4	3.6–7.3	18.1	14.0–22.1	24.8	16.1–33.5

^a^for the ten most frequently used pharmacological classes

### Determinants for the use of specific groups of medicines

Factors associated with the use of medicines may differ according to the type of medicine. Insight in this will help to understand better the association between those factors and polypharmacy. Therefore a multivariate analysis was performed considering drug groups at ATC_1 level (Table 4). Factors which are most associated with the use of all drug groups are having a longstanding illness, chronic conditions or handicaps, and having had at least one contact with a GP in past two months. A low education is associated with a higher use of drugs acting on the nervous system. For four of the six drug groups, a higher consumption is observed in the Walloon Region than in the other Regions.

**Table 4:** Determinants of use of specific categories of drugs - logistic regression (*N* = 2119)^b^, Belgium, 2008

Characteristic (reference category)	ATC C (*N* = 1387)		ATC N (*N* = 886)		ATC A (*N* = 734)		ATC B (*N* = 752)		ATC R (*N* = 245)		ATC M (*N* = 379)	
	OR	95 % CI	OR	95 % CI	OR	95 % CI	OR	95 % CI	OR	95 % CI	OR	95 % CI
Gender (male)												
Female	1.12	0.92–1.37	1.92^b^	1.58–2.34	1.44	1.18–1.76	0.61^b^	0.51–0.75	0.67^b^	0.50–0.90	1.18	0.93–1.50
Age (65–69)												
70–74	1.05	0.76–1.46	1.50^b^	1.06–2.11	1.15	0.82–1.61	1.50^b^	1.06–2.13	0.82	0.49–1.38	1.01	0.68–1.48
75–79	1.67^b^	1.20–2.34	1.32	0.93–1.88	0.96	0.67–1.35	1.80^b^	1.27–2.56	0.86	0.52–1.42	1.01	0.68–1.49
> = 80	1.45^b^	1.10–1.90	1.81^b^	1.35–2.42	1.05	0.79–1.40	2.36^b^	1.75–3.19	0.89	0.59–1.35	0.82	0.59–1.14
Education (Higher education)												
No degree/Primary	1.25	0.91–1.71	1.46^b^	1.07–1.98	1.17	0.86–1.58	1.33	0.98–1.81	1.23	0.78–1.93	1.25	0.87–1.79
Lower secondary	0.87	0.63–1.19	1.52^b^	1.12–2.08	0.92	0.67–1.26	1.16	0.85–1.59	1.22	0.77–1.95	1.36	0.95–1.96
Higher secondary	0.94	0.69–1.27	1.23	0.91–1.66	1.08	0.80–1.46	1.11	0.82–1.51	1.02	0.64–1.63	1.21	0.84–1.74
Equivalent household income (Quintile 5)												
Quintile 1	0.95	0.65–1.38	1.12	0.77–1.63	1.20	0.82–1.75	0.91	0.63–1.32	1.00	0.56–1.77	0.93	0.59–1.44
Quintile 2	1.13	0.79–1.61	1.01	0.71–1.43	1.18	0.82–1.70	1.03	0.73–1.47	1.37	0.80–2.34	1.04	0.68–1.58
Quintile 3	1.37	0.95–1.96	1.18	0.83–1.70	1.12	0.77–1.62	1.07	0.75–1.54	1.24	0.71–2.16	1.08	0.70–1.67
Quintile 4	0.98	0.66–1.46	1.18	0.79–1.75	1.04	0.69–1.55	1.09	0.73–1.62	1.09	0.59–2.02	1.10	0.68–1.77
Living situation (Living at home)												
Living in a residential home for the elderly	0.48^b^	0.33–0.71	1.25	0.84–1.86	1.40	0.96–2.04	0.74	0.50–1.10	1.15	0.69–1.42	0.64	0.37–1.11
Region (Flemish Region)												
Brussels Region	0.85	0.67–1.08	1.26	0.98–1.62	1.07	0.84–1.38	1.03	0.80–1.32	0.98	0.68–1.42	1.29	0.96–1.73
Walloon Region	1.76^b^	1.40–2.21	1.55^b^	1.25–1.93	1.51^b^	1.22–1.88	1.27^b^	1.02–1.57	0.99	0.72–1.37	1.23	0.95–1.61
Depression < 12 months (no)												
Yes	0.77	0.52–1.13	4.76^b^	2.97–7.64	1.51^b^	1.06–2.17	0.77	0.52–1.14	1.55	0.97–2.48	0.70	0.43–1.16
Longstanding disease, condition or handicap (no)												
Yes	1.42^b^	1.16–1.73	1.90^b^	1.56–2.39	2.18^b^	1.78–2.67	1.65^b^	1.35–2.02	2.74^b^	1.96–3.83	1.66^b^	1.30–2.14
Contact with GP in past 2 months (no)												
Yes	2.90^b^	2.31–3.66	1.86^b^	1.45–2.39	1.51^b^	1.17–1.96	1.86^b^	1.43–2.40	2.45^b^	1.54–3.90	1.18	0.87–1.60
Ambulatory contact with specialist in past 2 months (no)												
Yes	1.14	0.91–1.44	1.07	0.86–1.33	1.37^b^	1.11–1.71	1.35^b^	1.09–1.69	1.21	0.89–1.63	1.25	0.96–1.62
Inpatient hospitalization in past 12 months (no)												
Yes	1.46^b^	1.13–1.91	1.52^b^	1.20–1.94	1.32^b^	1.04–1.68	1.43^b^	1.13–1.81	1.38^b^	1.01–1.88	0.85	0.63–1.14
Day patient hospitalization in past 12 months (no)												
Yes	0.86	0.62–1.19	1.13	0.82–1.56	0.97	0.71–1.34	1.20	0.87–1.65	0.80	0.49–1.30	0.80	0.54–1.20

^b^all the factors in the table are included in the model

People living in institutions for older people use less cardiovascular medicines than those living at home (OR: 0.48). An analysis performed at ATC_2 level shows that three groups of medicines active on the cardiovascular system are significantly less frequently used in institutions: lipid modifying agents, beta blockers and agents acting on the renin-angiotensin system.

### Determinants of polypharmacy

The multinomial logistic regression shows that the socio-demographic factors which are most associated with EPP are as follows (Table 5): living in the Walloon Region (OR: 2.57) and being aged ≥80 years (OR: 2.08); they are associated to a lesser extent to polypharmacy. Having no degree or a primary education is more associated with polypharmacy (OR: 1.52; 95 % CI: 1.07–2.15) than with EPP (OR: 1.32; 95 % CI: 0.75–2.31).

**Table 5:** Determinants of polypharmacy - multinomial logistic regression (reference 0–4 medicines; *N* = 2119), Belgium, 2008

Characteristic	5–8 medicines (*N* = 617)		> = 9 medicines (*N* = 181)	
	OR	95 % CI	OR	95 % CI
Gender				
Males	1		1	
Females	1.13	(0.91–1.40)	1.19	(0.83–1.71)
Age				
65–69	1		1	
70–74	1.30	(0.89–1.92)	1.51	(0.74–3.06)
75–79	1.38	(0.94–2.02)	1.69	(0.86–3.34)
> = 80	1.78^a^	(1.29–2.45)	2.08^a^	(1.15–3.77)
Education				
No degree/Primary	1.52^a^	(1.07–2.15)	1.32	(0.75–2.31)
Lower secondary	1.19	(0.83–1.71)	1.17	(0.64–2.13)
Higher secondary	1.18	(0.84–1.67)	1.19	(0.66–2.13)
Higher education	1		1	
Equivalent household income in quintiles				
1	0.95	(0.63–1.45)	1.62	(0.79–3.31)
2	1.05	(0.70–1.55)	1.31	(0.66–2.60)
3	1.22	(0.82–1.83)	1.41	(0.68–2.90)
4	0.88	(0.56–1.39)	1.20	(0.55–2.62)
5	1		1	
Living situation				
Living at home	1		1	
Living in a residential home for the elderly	1.09	(0.71–1.65)	1.68	(0.92–3.05)
Region				
Flemish Region	1		1	
Brussels Region	1.06	(0.81–1.39)	1.08	(0.66–1.77)
Walloon Region	1.48^a^	(1.17–1.88)	2.57^a^	(1.71–3.85)
Depression < 12 months				
Yes	1.54^a^	(1.02–2.33)	3.48^a^	(2.03–5.97)
No	1		1	
Longstanding disease, condition or handicap				
Yes	2.45^a^	(1.97–3.05)	5.67^a^	(3.58–8.98)
No	1		1	
Contact with GP in past 2 months				
Yes	2.92^a^	(2.14–3.98)	4.72^a^	(2.32–9.61)
No	1		1	
Ambulatory contact with specialist in past 2 months				
Yes	1.19	(0.93–1.51)	1.58^a^	(1.09–2.28)
No	1		1	
Inpatient hospitalization in past 12 months				
Yes	1.36^a^	(1.05–1.77)	2.20^a^	(1.51–3.20)
No	1		1	
Day patient hospitalization in past 12 months				
Yes	1.04	(0.74–1.47)	1.07	(0.62–1.86)
No	1		1	

^a^differences statistically significant

For EPP-users, the highest odds ratio is found for the group having the lowest equivalent household income, but differences are statistically not significant and the confidence intervals are very wide; no conclusion should be drawn.

The analysis shows that the health related factors which are most associated with EPP are as follows : having a longstanding illness, chronic conditions or handicaps, at least one contact with a GP in past two months, a self-reported depression during the last year. Those factors are also associated with polypharmacy but to a lesser extent. At least one inpatient hospitalization in past 12 months is also associated with EPP and to a lesser extent with polypharmacy.

## Discussion

In the present study we investigated correlates of (excessive) polypharmacy in a nationwide representative sample in Belgium, taking into account socio-demographic information, as well as the health status of the individuals and the corresponding use of health care.

Excessive polypharmacy was defined here as the use of nine different medicines or more in the past 24 h, and not ten medicines or more as suggested in some cases. The reason for this choice is related to the fact that the products considered here were limited to official medicines listed in the Annotated Directory of Medicines published by the BCPI. The expression “excessive polypharmacy” (EPP) refers only to the concomitant use of at least nine different medicines, but has no negative connotation here in terms of inappropriate polypharmacy. Indeed, by simply counting the number of different medicines it is not possible to assess the appropriateness of medicine use. Inappropriate medicine use is far more difficult to determine and out of the scope of the present study.

The medicines considered here include all pharmaceutical products used in the past 24 h, either on a regular basis either occasionally; no information is available on the indication, the duration of the therapy and the dosage.

EPP can be due to treatment of co-existing diseases or to the use of different medicines for the same disease. Polypharmacy is facilitated by the increase in the number of medicines available on the market and an easier access to medicines as a result of the exponential growth of the generic medicine market.

### Comparisons between the results from this study and those from previous studies

Comparisons with other studies are difficult due to different definitions of EPP, countries, study years (more and more new medicines are produced and made available), settings (population or institution based), study design, sampling and data collection methods. The varying definition of a medicinal product and reference periods is also a difficulty: some studies, for instance, take only into account prescription medicines (sometimes even only the reimbursed ones), while others include over-the-counter medicines, vitamins and mineral supplements, herbal products; some take only into account routinely administered medicines, excluding agents only used when needed (“as-needed”).

Factors related to the health care system can also induce differences between countries: organizational characteristics, availability of medicines on the market, country specific regulatory medicines measures: prescription status, reimbursement system (subsidized versus non-subsidized medicines, level of patient co-payment), prescribing attitudes.

Several medicines may be consumed weekly, monthly or for short periods. Longer assessment periods tend to find higher prevalence rates [4]. In 2006 in Sweden [16], the prevalence of five or more different prescription drugs dispensed was 11.3, 17.2 and 24.4 % when the study period was 3, 6 and 12 months respectively.

Variations in prevalence rates of EPP between studies may also be related to differences in the population that is studied, as it has been shown by Fialová et al. [4] among older home care patients in Europe, in which differences among the eight participating countries were significant for all study population characteristics.

For all these reasons, the comparison of the results of the present study with previous studies, was restricted to studies performed in European countries from 1998 onwards.

In the present study, one fourth of the people aged 65 years or more belongs to the *polypharmacy group* and 8.2 % to the *EPP-group*. In a study of older patients receiving home care enrolled in metropolitan areas of eight countries in Europe in 2001–2002 [4], the 7-day prevalence of medication use was evaluated: excessive polypharmacy (≥9 medications) was documented in 22.2 % of the patients, varying between 7.0 and 41.2 % of patients according to the country. In 1998 in the Kuopio 75+ study (Finland), 22.7 % of persons living at home used ten or more medicines concomitantly [12].

When considering only the people living in a residential home for the elderly, the mean *number of medicines used per person* is 4.5 (range: 0–18) and the prevalence of EPP is 16.8 %. This figure falls within the range of the results found in an European study conducted from 2009 to 2011 in nursing homes in eight countries: 8.8–56.7 % excessive polypharmacy (≥10 medications in the 3 days prior the assessment) [10]. In 2003, Pitruzzella et al. [6] found in institutions for aged people in the Walloon Region (Belgium), that 18.6 % of the residents received more than 10 medicines on one day.

In 2005, a large representative sample of residents of Belgian nursing homes has been investigated: they had a mean of 8.4 prescriptions (range: 0–22), one-third had at least 10 medications lines noted on their medication chart and only 1 % had no medication [8]. The higher percentage of EPP in the this last study could be related to the reference periods used (medicines taken in the 24 h prior to the interview in the present study versus “lines on the medication chart”, which does not mean 24 h use), a selection bias in the health interview survey (representativity of the respondents in nursing homes: participating respondents may be in better health) and the fact that use of medications not frequently used is probably underestimated in our survey [17].

A multivariate analysis was performed to study the *factors associated* with (excessive) polypharmacy. Jyrkkä et al. also investigated both polypharmacy and excessive polypharmacy, but polypharmacy was defined as the use of 6 to 9 drugs concomitantly and excessive polypharmacy as the use of 10 or more drugs [12].

Previous studies (see details below) on factors associated with (excessive) polypharmacy give conflicting results for gender, age, socio-economic status and living or not in an institution for elderly people. For depression, primary care visits and inpatient hospitalization, studies are in agreement and our results are in line with those from previous studies.

*Female gender* is not associated in the present study with polypharmacy nor EPP. The same was observed in out-patient prescriptions in 1994 in Sweden [18]. However two studies showed an association: in the non-institutionalized elderly in Castile-Leon (Spain) in 2006 [13, 16] and only for excessive polypharmacy in 1998 in the Kuopio 75+ study (Finland) [12].

Being *age*d ≥80 years is associated in the present study with EPP and to a lesser extent to polypharmacy. Some studies have shown an association only between age ≥85 years and excessive polypharmacy [12], while others found in nursing homes in Europe (2009–2011) increasing age associated with a reduced rate of excessive polypharmacy [10].

Having no degree or only a primary education *degree* is associated in the present study with polypharmacy. Having an education level lower than higher secondary is the most associated with the consumption of drugs acting on the nervous system. Haider et al. [19] observed in Sweden in 2002 among people aged ≥77 years that the association between low education level and polypharmacy was not significant (after adjustment for age, sex, comorbidity, marital status, and living situation).

*Living in an institution for the elderly* is associated in the present study with both polypharmacy and EPP, but the association it is not statistically significant. Haider et al. [19] observed a higher prevalence of polypharmacy for older people living in institutions than for the community-dwelling elderly. In a study of the Belgian socialist mutuality - Solidaris, the consumption of reimbursed medicines was followed in 2009–2011 in patients aged 70 and over, six months before and six months after their entry into nursing home. Institutionalization had no impact on the number of different medicines used by the older people. On average, patients consumed 8 different molecules and that number was the same six months before and six months after the entry into nursing home [20]. In 2011, a study of the Belgian national union of independent health insurance funds groups on reimbursed medicines dispensed to their members residing in nursing homes also showed that polypharmacy remains virtually unchanged following institutionalization [21].

*Living in the Walloon Region* is associated in the present study with both polypharmacy and excessive polypharmacy. The multivariate analysis performed on drug groups at ATC_1 level showed that four of the six drug groups considered are more consumed in the Walloon Region than in the other Regions of residence. This could reflect prescription habits and could also be related to the higher percentage of people in Brussels and Walloon Regions who reported suffering from at least one chronic or long-term disease or handicap^2^.

A self-reported *depression* during the last year is associated in the present study with (excessive) polypharmacy. The same was observed in 1998 in the Kuopio 75+ study [12] as well as in an European study conducted from 2009 to 2011 in nursing homes [10].

As can be expected, *longstanding illness, chronic conditions or handicaps* is also associated in the present study with both polypharmacy and EPP. As this information is not mentioned in other studies, we looked at bad subjective health as a proxy. In the non-institutionalized older people in Castile-Leõn (Spain) in 2006, regular poor self-perceived health was associated with polypharmacy [13]. In 1997–1999, self-reported poor health correlated with increasing number of drugs taken by 70–74 year old community dwelling individuals in Western Norway [22]. In 1998 in the Kuopio 75+ study, moderate self-reported health was associated only with excessive polypharmacy, whereas poor self-reported health was associated with both polypharmacy and excessive polypharmacy [12]. Also in the 1998–1999 Lieto study, persons with polypharmacy had poor self-reported health [23].

At least one *contact with a general practitioner* in past two months is associated in the present study with both polypharmacy and EPP while the contacts with a specialist is associated only with EPP. Jörgensen et al. [18] found in 1994 in Sweden a relationship between the number of primary care visits and multiple prescription drug use (5 or more different drugs during one year).

At least one *inpatient hospitalization* in past 12 months is associated here with both polypharmacy and EPP, while no relation is found for day patient hospitalization. Jörgensen et al. [18] found also a relationship between hospitalization during the year and multiple prescription drug use.

Medicines active on the cardiovascular system are the *most frequently used drugs*, followed by the drugs acting on the nervous system. This is also the case in the Lieto study [23] and the Kuopio75+ study [12]. In 2005, in a sample of residents of Belgian nursing homes, the drugs acting on the nervous system were the most frequently used, followed by those acting on the digestive tract and on the metabolism, and on the cardiovascular system [8]. In 2011, a study of the Belgian national union of independent health insurance funds groups on reimbursed medicines dispensed to their members residing in nursing homes found that the drugs acting on the nervous system were the most frequently used, followed by the anti-infective agents for systemic use and the cardiovascular medicines [21].

At the ATC_3 level, the most frequently used drugs in the current study are antithrombotic agents. This has also been found in Sweden in 2002 [19]. They are followed by lipid modifying agents (plain), β-blocking agents, and drugs for peptic ulcer and gastro-oesophageal reflux disease. In 2012, a study of medicines reimbursed in Belgium [24] found also the same four most frequently used drugs associated with polypharmacy. When we consider only people in the polypharmacy or EPP groups, the same is found in the present study, even if the ranking is not exactly the same.

### Strengths and limitations of the present study

This study as some *strengths*. It is a population-based study including a representative sample of the general population living at home and in institutions for the elderly. The information about medicine use, prescribed and OTC medicines, was based on the respondents self-reports; the information displayed on their medicine packages was also checked to “validate” the information. Topical treatments were included in the medicines considered because they may also have systemic effects and consequently increase the risks of interaction [25, 26]

The Health Interview Survey collects information during a whole year, allowing to control the seasonal effect on medicines consumption; it is also allowing to get a fairly accurate estimate of the number of users of a specific drug on an average day of the year. Information was gathered on the use of medicines taken in the 24 h prior to the interview; the recall bias should consequently be minimal. In addition, information was collected on health status and health determinants, which allows to study factors associated with medicine consumption.

In the present study also non-prescribed and non-reimbursed medicinal products are included. In prescription database studies, non-prescribed (OTC medicines) and sometimes also non-reimbursed medicines (medicines not subsidized by the health insurance system) are excluded. Such studies allow only to estimate dispensed medicines, which may differ from the actual consumption (not all medicines purchased are used and some medicines can be used a long time after their purchase).

In 2008 in Belgium, 50 % of out-patient market by volume are reimbursable medicines, 38 % are non-reimbursable OTC medicines and 12 % are other non-reimbursable medicines (generally subject to medical prescription)^3^. In the present survey, 18 % of the elderly population reporting having taken at least one prescription medicine over the previous two weeks took also at least one OTC medicine.

There are also some *limitations* in the present study. First, since the use of medicines not listed in the Annotated Directory of Medicines has not been considered - such as magistral preparations, herbal products, natural supplements^4^, homeopathic medicines -, the incidence of excessive polypharmacy is probably higher than the one calculated here. Another possible cause of underestimation could be related to the fact that some medicines classified as one product may in fact contain more than one chemical entity; medicine users may thus wrongly be classified as non-(excessive) polypharmacy users. The short recall time period used here (24 h) may also result in an underestimation for the medicines that are not used daily, but rather weekly or even monthly.

The second limitation is related to the fact that the health status, the medical diagnosis and the number of contacts with a health professional are self-reported. Third, the non-respondents could have a worse health status and use more medicines than the respondents [22]. Unfortunately, health related information on the characteristics of the non-participants, both persons who were non-contactable and persons who refused to participate, is lacking. A study however showed that the presence of at least one household member with a bad self-perceived health has a negative impact on participation [27].

In Europe, few population based health surveys include questions regarding the consumption of all medicines (prescribed and OTC) during the last 24 h; when they do, they are limited to a city [12], or a county [22]. In France, questions about consumption of medicines the day before the survey have been included from 1998 till 2010 in the “Enquête sur la Santé et la Protection Sociale”, but have been removed in 2012 [28]. In 2006 in Greece, a study excluded individuals reporting only OTC drug use [29]. In the Lieto study among community-dwelling persons aged 64 years or over, prescription drugs during 7 days prior to the interview was recorded [23]. In Barcelona, the use of 13 classes of prescribed and non-prescribed drugs in the two weeks prior to the interview was registered in a non-institutionalized population [30]. In the European Health Interview Survey, which targets the population aged at least 15 and living in private households, consumption of prescribed and non-prescribed drugs in the two weeks prior to the interview is recorded (but no information is available on the type of drug). In Spain, consumption by the non-institutionalized population of prescribed and non-prescribed drugs in the two weeks prior to the interview is recorded [13].

## Conclusions

As older people are a growing population and there is also an increase in the availability of medicines on the market, excessive polypharmacy could become an escalating public health problem in the coming years.

In order to optimize drug use, it is necessary to find a balance between adequate treatment of diseases and avoiding adverse drug effects. The risks can be minimized by increasing awareness among healthcare professionals and patients. A periodic review of the patient’s medicine list that contains all prescribed but also OTC medicines is necessary to readjust drug treatment and avoid inappropriate excessive polypharmacy which could have harmful effects. Physicians have to balance the benefits and risks related to multiple medicine use and to avoid “prescribing cascades”.

When limiting the number of different medicines taken, attention should be paid by the physicians to the danger of under-prescription of beneficial medicines because the probability of under-prescription increases with the number of drugs used [31].

The involvement of patients is also an important factor. As 94 % of persons in the EPP-group had at least one contact with a GP in past two months, this task of review and coordination between prescribers is one that should be fulfilled by GPs. Since more than 90 % of persons in the “(excessive) polypharmacy” group use cardiovascular medicines, interventions could be focused on patients with cardiovascular diseases as suggested in some studies and mentioned by Jörgensen et al. [18], but also on other risk groups such as those suffering from depression and those aged 80 years and over. National campaigns to raise awareness of prescribers and patients about the dangers related to excessive polypharmacy should be organized.

In order to reduce polypharmacy, some countries published recommendations, like “a Polypharmacy Guidance for 2012” by NHS Scotland [32], “Prescribing for older people” by the Welsh Medicines Resource Centre [33], or the guidelines published by the Dutch College of General Practitioners [34]. In Belgium, EBMPracticeNet (Evidence-Based Medicine) published EBM Guidelines^5^ in 2010, and the Farmaka association published in 2013 and in 2014 a memento and also newsletters related to the topic^6^. This is one of the strategies which could help to optimize drug therapy, to further develop.

The periodical health surveys allow to study the prevalence of medicine use and the “true” concomitant exposure to medicines, to study its evolution and the associated factors. They provide complementary information when compared to medicine utilization studies based on sales or prescriptions. The situation must be monitored closely and studied more in depth in order to identify the most important determinants of excessive polypharmacy.

## Abbreviations

ATC: Anatomical Therapeutic Chemical classification system of the World Health Organization
BCPI: Belgian Centre for Pharmacotherapeutic Information
DDD: Defined Daily Dose
EPP: Excessive polypharmacy
GP: General practitioner
OTC: Over-the-counter medicines

## Footnotes

1 http://www.whocc.no/atc_ddd_index/ World Health Organization Collaborating Centre. (2009) *Use of ATC/DDD*. (Updated 10 December 2009)

2 https://hisia.wiv-isp.be/SitePages/Home.aspx

3 http://www.pharma.be/assets/files/2178/2178_130361752513134000.pdf paper downloaded on 14/8/2014 "Marché des spécialités pharmaceutiques en Belgique (ambulatoire/hospitalier)"

4 CBIP: Only preparations registered as medicinal preparations are mentioned here. Many preparations made of minerals and vitamins are available as dietary supplements. Vitamins are officially considered as food ("nutrients") when the recommended daily dose is approximately the daily needs.

5 https://www.ebmpracticenet.be//fr/Pages/default.aspx?ebmid=ebm00468&sn=38df8045-613d-4b16-a4ed-683e9d22b5f0/31f15519-99f5-4c53-9e1e-62b6f0099736

6 http://www.farmaka.be
